# A national population-based assessment of 2007–2008 election-related violence in Kenya

**DOI:** 10.1186/1752-1505-8-2

**Published:** 2014-01-18

**Authors:** Kirsten Johnson, Jennifer Scott, Treny Sasyniuk, David Ndetei, Michael Kisielewski, Shada Rouhani, Susan Bartels, Victoria Mutiso, Anne Mbwayo, David Rae, Lynn Lawry

**Affiliations:** 1 The Department of Family Medicine, McGill University, Montreal QC, Canada; 2 Division of Women’s Health, Brigham and Women’s Hospital, Boston MA, USA; 3 Department of Obstetrics & Gynecology, Beth Israel Deaconess Medical Center, Boston MA, USA; 4 University of Nairobi, Nairobi, Kenya; 5 Africa Mental Health Foundation, Nairobi, Kenya; 6 Lawry Research Associates International, Waldorf, MD, USA; 7 Department of Emergency Medicine, Brigham and Women’s Hospital, Boston, MA, USA; 8 Department of Emergency Medicine, Beth Israel Deaconess Medical Center, Boston, MA, USA; 9 Department of Statistics, American University, Washington, DC, USA; 10 American Sociological Association, Washington, DC, USA

**Keywords:** Kenya 2007 elections, Election violence, Politically-motivated sexual violence, Mental health, Human rights violations

## Abstract

**Background:**

Following the contested national elections in 2007, violence occurred throughout Kenya. The objective of this study was to assess the prevalence, characteristics, and health consequences of the 2007–2008 election-related violence.

**Methods:**

A cross-sectional, national, population-based cluster survey of 956 Kenyan adults aged ≥ 18 years was conducted in Kenya in September 2011 utilizing a two-stage 90 x 10 cluster sample design and structured interviews and questionnaires. Prevalence of all forms of violence surrounding the 2007 election period, symptoms of major depressive disorder (MDD) and posttraumatic stress disorder (PTSD), and morbidity related to sexual and physical violence were assessed.

**Results:**

Of 956 households surveyed, 916 households participated (response rate 95.8%). Compared to pre-election, election-related sexual violence incidents/1000 persons/year increased over 60-fold (39.1-2370.1; p < .001) with a concurrent 37-fold increase in opportunistic sexual violence (5.2-183.1; p < .001). Physical and other human rights violations increased 80-fold (25.0-1987.1; p < .001) compared to pre-election. Overall, 50% of households reported at least one physical or sexual violation. Households reporting violence were more likely to report violence among female household members (66.6% vs. 58.1%; p = .04) or among the Luhya ethnic group (17.0% vs. 13.8%; p = 0.03). The most common perpetrators of election-related sexual violence were reported to be affiliated with government or political groups (1670.5 incidents/1000 persons per year); the Kalenjin ethnic group for physical violations (54.6%). Over thirty percent of respondents met MDD and PTSD symptom criteria; however, symptoms of MDD (females, 63.3%; males, 36.7%; p = .01) and suicidal ideation (females, 68.5%; males, 31.5%; p = .04) were more common among females. Substance abuse was more common among males (males, 71.2%; females, 28.8%; p < .001).

**Conclusion:**

On a national level in Kenya, politically-motivated and opportunistic sexual and physical violations were commonly reported among sampled adults with associated health and mental health outcomes.

## Background

Following the contested Republic of Kenya General Election on 27 December 2007, violence erupted throughout the country after the announcement on 30 December 2007 that the Party of National Unity (PNU) headed by Mwai Kibaki had defeated the Orange Democratic Movement (ODM) led by Raila Odinga. In Kenya, political affiliations tend to follow ethnic lines and most of the attacks were reported to be carried out by Kalenjin pro-government PNU supporters [1-3]. Nonetheless, widespread violence targeted against both PNU and ODM supporters was reported, including murder, physical and sexual violence, loss of property, and forced displacement [1-3]. The official number of deaths reported was 1,133 [3].

The election violence lasted for 59 days, from 28 December 2007 until 28 February 2008, when a power-sharing agreement naming Kibaki as President and Odinga as Prime Minister was signed [4,5]. The power-sharing agreement, called the National Accord and Reconciliation Act, established the coalition government—in addition to four main agenda items—to end the political crisis and address its underlying causes. The Truth, Justice and Reconciliation Commission of Kenya (TJRC) was part of the accountability component of the fourth agenda item in the National Accord [6,7].

Following the election violence, the Waki Commission and a special tribunal were also established to investigate and hold accountable those involved in election-related violent crimes [4]. This tribunal—in addition to other national and international efforts—led to indictments alleging crimes against humanity [8]. In December 2010, the International Criminal Court (ICC) Prosecutor requested that six individuals be summoned to appear before the Court in two separate cases. Charges against two of them, Henry Kiprono Kosgey and Muhammed Hussein Ali, were rejected by Pre-Trial Chamber II on 23 January 2012. Case one now involves two members of the ODM—the opposition party at the time of the elections—and case two involves two members of the PNU, then the incumbent party. The Prosecutor has brought charges against William Ruto and Joshua Arap Sang, members of the ODM at the time of violence, for the crimes against humanity of murder, forcible transfer, and persecution—allegedly committed against PNU supporters. The Prosecutor has brought charges against the current President, Uhuru Kenyatta—a member of the PNU at the time of the violence—for the crimes against humanity of murder, forcible transfer, rape, persecution, and other inhumane acts allegedly committed against ODM supporters, partly in retaliation to attacks against the PNU supporters [9].

Since the 2007–2008 election violence, healthcare centers, nongovernmental organizations, and advocacy groups amongst others have provided care for survivors of the violence, created platforms to document election-related sexual violence and other human rights violations, in addition to promoting human rights awareness [3,10]. Political and healthcare reforms have been introduced and represent positive change in the aftermath of the crisis [6,7,11-13].

Kenya’s most recent presidential elections in March 2013 did not exhibit the same scale of violence as the preceding elections, but violence still occurred prior to the elections, claimed over 400 lives, and displaced an estimated 118,000 persons. One of the factors cited as an underlying cause of the 2013 election-related violence was a lack of justice from the 2007–2008 post-election violence [14]. Although Kenyans averted a recurrence of the 2007–2008 post-election violence, the conflict drivers that triggered the violence, including a culture of impunity, land grievances, corruption, ethnic tensions, weak institutions, and regional and socioeconomic inequality, have yet to be addressed adequately [15].

Studies have documented the health and mental health outcomes of sexual violence and human rights abuses in populations affected by internal conflict [3,16-18], but few have examined politically-motivated sexual violence and its associated physical and mental health consequences. This study applies a well-established methodology to gather population-based information on politically-motivated violence and its health and mental health consequences to better understand the needs of survivors and to inform programming and policy at local and national levels. These data provide a more precise understanding of the Kenyan post-election violence from 2007–2008.

## Methods

### Survey sites and sample selection

**Figure 1:**
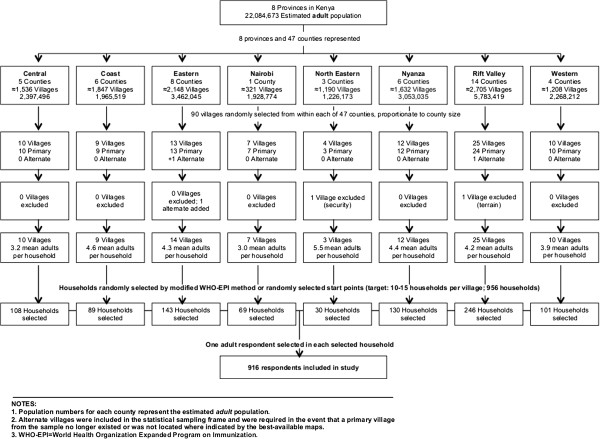
Flow of the study sample.

A national, cross-sectional study was conducted in Kenya over four weeks in September 2011. Systematic sampling included a 90 × 10 (90 villages × 10 households) cluster-based sampling frame with a total of 916 households included in the final sample, representing 22.1 million adults in eight provinces and 47 counties. (Figure 1).

Based on our research in other conflict and post-conflict settings and because the true prevalence of depression was unknown in the Kenyan context, we chose a conservative estimate of 50% for the prevalence of depression in order to calculate sample size [19-22]. A sample size of 900 households was required to estimate that prevalence via a simple random sample to within 0.05 with 95% confidence, and to account for a design effect of “2” and refusals [23,24]. Two of the 90 villages required a substitution: one for security reasons and one due to difficult roads and geography despite several attempts to access the village. (Figure 1).

Population estimates were obtained from 2009 national census data [25]. The sampling plan included stratification by all eight provinces and 47 corresponding counties according to population size. Stratification was performed by geographic area by sampling proportionally to population size [11]. Using a random number generator, villages within counties were chosen from a master data file provided to the researchers by the Bureau of Statistics. Because village population data were unavailable, each survey team collected village-level population estimates at the time of the survey from village elders, to create adult-person and household-level statistical weights for the analysis.

Households were sampled at the village level using a modified WHO EPI Method [20,26,27]. Interviewers began in the geographic center of the village and used a pen to determine the direction in which to proceed [24]. The number of houses to pass to reach the first sample unit (and subsequent sampling interval) was determined randomly by drawing a number from one through 10 from an envelope. Ten to 15 households per village were sampled. (Figure 1) One adult (≥18 years) who had resided in the house for at least the past three months was randomly selected and interviewed per household. Prior to entering the household, the interviewer tossed a coin to randomly select the sex of the respondent; the interviewer then requested the interview according to the sex selected. If multiple people in one household were eligible, a table listing age (oldest to youngest) was used to randomly select a respondent. If only one adult household member or only one male or female was present at the time of the survey, that person was interviewed. Records were kept of refusals, ineligible households, and lack of availability after two attempts.

One-on-one interviews were conducted anonymously in a setting that offered privacy and confidentiality, typically inside the housing unit. Informed consent was obtained and respondents were given the opportunity to opt out of the survey at any time. Respondents were provided with a referral to local nongovernmental organizations and health centers that offered mental health programs if distress was noted or expressed [26].

### Instrument

The survey contained 115 questions. Respondents were asked to report on personal and household demographics, basic needs, income, education, healthcare access, morbidity, and physical, sexual, and movement violations with regard to pre- (before the 2007 election), 2007 election-related (December 2007-March 2008), and post-election (March 2008 to survey administration). Respondents were asked to self-report on physical and mental health symptoms and substance use, and to provide their opinions regarding elections in Kenya [20,21,26,28-31]. A 10-year recall of events was considered reliable [32].

A household was defined as the group of people eating and sleeping under the same roof. Substance abuse was noted if the participant reported using drugs or alcohol on a regular basis; more than two times per week or in excess each time [33]. Inadequate general healthcare was determined if respondents reported that medical services either were unavailable to them, were too far away, or that they lacked transportation to those services.

Respondents were asked about lifetime experiences of violence. Intimate partner violence (IPV) included physical violence, such as beatings by a spouse or partner, and sexual violence. Sexual violence was defined as any violence—physical or psychological—carried out through sexual means or by targeting sexuality, and included rape and attempted rape, molestation, sexual slavery, being forced to undress or being stripped of clothing, forced marriage, insertion of foreign objects into the genital opening or anus, forcing two victims to perform sexual acts on one another or harm one another in a sexual manner, or mutilating a person’s genitals [34]. Gang rape was defined as rape by two or more individuals [34].

A perpetrator was defined as any person who directly inflicted violence or a reported abuse [34]. Human rights violations included physical violations (beating, shooting, stabbing, amputation, or other physical assault), sexual violence, theft or destruction of property, abduction, detainment, movement violations (being forced to leave the home due to fear or direct threat), forced labor, and forced use of drugs [26]. Politically-motivated violations were defined as any violations perpetrated by a political leader or state official (e.g., police officer) that occurred in Kenya during the period of December 2007 to March 2008, and violations that included ethnic slurs or political messages uttered during the attack. Opportunistic violence was defined as any form of violence that did not meet the criteria for politically-motivated violence.

The PTSD Symptom Scale Interview (PSS-I) was used to assess symptoms of posttraumatic stress disorder (PTSD), which has an 86% sensitivity and 78% specificity for PTSD using a one-month recall period [35]. Symptoms of major depressive disorder (MDD) were assessed using the *Patient Health Questionnaire–9*, a well-validated, highly sensitive instrument for identifying individuals with current and past depression [36]. Although not validated specifically for post-conflict settings, the PTSD scale has been reliably used in communities in post-conflict settings in Africa including Uganda [37], Rwanda [22], Liberia [20], and Democratic Republic of Congo (DRC) [26], and the MDD scale in Sudan [21], Sierra Leone [30], Liberia [20], and DRC [26]. The PTSD symptoms variable was set to “1” if the respondent met the *Diagnostic and Statistical Manual of Mental Disorders (Fourth Edition DSM-IV*) criteria based on their answers to the PSS-I portion of the interview [26,33,35]. The MDD variable was set to “1” if the respondent met the *DSM-IV* criteria based on their answers to the *Patient Health Questionnaire–9* portion of the interview [33]. Questions about suicidal ideation [38] during the previous year and lifetime suicide attempts [39] were answered with a “yes” or “no”. Mental health counseling was defined as “having someone to talk to about your problems that will listen and give emotional support”.

Respondents were asked the number of household members who died as a result of the post-election violence. To assess physical, sexual, and movement violations, respondents were asked whether they or their household members had been beaten, shot, stabbed, seriously injured, sexually assaulted, raped, abducted, had violent amputations and/or circumcision, were forcibly displaced, or suffered property damage. For each violation, respondents were asked the sex of the abused, type of abuse, whom they thought committed the violation, the identity and sex of the perpetrator, the number of attackers, and the consequences [40].

The survey was written in English, translated into Kiswahili by a Kenyan fluent in English and Swahili, back-translated by interviewers into English and administered in Kiswahili. Kenyan interviewers and researchers reviewed the survey for clarity and cultural appropriateness. The survey was pilot-tested to establish clarity of questions and for cultural appropriateness among residents in the Kibera area of Nairobi [41], and minor, appropriate changes were incorporated into the final survey.

### Interviewers

Twenty-one Kenyan interviewers (7 males; 14 females) from different ethnic groups were trained by five expatriate and four Kenyan researchers, involving classroom-based teaching and experimental role-play on topics including completion of the survey, sampling techniques, informed consent, logistics, confidentiality, sexual violence, and mental health. The final day of the training included a pilot test.

Interviewers were not matched by sex to respondents and were placed into groups of two or three depending on security and distance required to travel. Interviewers met with study researchers at a central location at the end of each study day to review all surveys for completion, and they maintained contact via mobile phone to answer questions and monitor progress. Interviewers were able to complete an average of three to five surveys per day and worked between 10 and 14 days.

### Human subjects approval

Ethics approval was obtained from Kenyatta National Hospital/University of Nairobi, McGill University, and Brigham and Women’s Hospital (BWH) at Harvard Medical School. Permission to conduct research in Kenya was obtained from the National Council for Science and Technology. Interviewers were hired through the study’s partner organization, the Africa Mental Health Foundation. Informed consent was obtained from all participants. As required by the Kenyatta Institutional Review Board (verbal consent was approved and written consent waived by McGill University and BWH), a record of the respondent’s consent was obtained by either an ink thumbprint or an unidentifiable mark placed on a cover sheet kept separate from the survey. Every effort was made to ensure protection and confidentiality and to reduce any potential adverse consequences to participants. Respondents did not receive material compensation. They were informed that participation or lack thereof would not affect their access to or the quality of the care that they receive and were explicitly given the right to refuse participation.

### Statistical analysis

Data analysis was conducted in R (Version 2.14.0), an open-source version of S-PLUS. Data were weighted according to the proportion of the adult population living at the provincial, county, and (estimated) village level, such that the adult person weights sum to the adult Kenyan population as of the 2009 national census. The data were further weighted to adjust for the difference in sex ratio between the final sample and the sex ratio for the adult Kenyan population, using county- and provincial-level sex ratio data.

To enable a comparison of sexual violations and human rights violations data between the pre-2007 election (baseline), election violence, and post-election violence periods, weights were applied to create mean occurrences (of a violation) per 1,000 persons per year. Time periods were created as such: three and one-half years from administration of the survey in September 2011 to after the election violence ended in February 2008, 59 days for the election violence period (end of December 2007–February 28, 2008), and six years for the pre-election period (from the end of December 2007 backwards). To allow for comparison, the pre-election period is reduced by a factor of 0.160, the election violence period expanded by a factor of 6.083, and the post-election violence period reduced by a factor of 0.278 [42]. *P*-values were calculated between the pre-election time period and the election violence time period using a pairwise test with P < .05 considered significant, a two-sample t-test for the difference between two proportions for the mental health data, and a Wald test of association for all other *P*-values.

## Results

### Demographics

Of the 956 households sampled, 916 respondents completed the survey, for a response rate of 95.8%: 23 declined to participate (nine due to lack of time, three due to opposition to study, and 11 for other, unspecified reasons); two were unavailable after two visits; and 15 consented but did not complete the interview (one due to interruption, two due to safety concerns, one requested to stop; 11 for other, unspecified reasons). (Figure 1).

Altogether, 549 females and 367 males were interviewed, with a mean age of 37.7 years and an average household size of 6.9 persons. (Table 1) The majority of respondents were married (70.8%) and Christian (87.6%), and the most frequently reported ethnic groups were Kikuyu (21.8%) and Luhya (13.8%). Households reporting violence were more likely to report violence among female household members (66.6% vs. 58.1%; p = .04) or among the Luhya ethnic group (17.0% vs. 13.8%; p = 0.03). Eleven percent of respondents lost land as a result of the election. Of 566 respondents, 13.6% had their schooling or vocational training interrupted due to the election violence; of those whose schooling/training was interrupted, 85.5% were able to return to their training after the violence had ended.

**Table 1:** Weighted population characteristics: Kenyan adult household-based population (916 respondents)

	**Weighted %**^**a **^**(95% CI)**	
**Characteristic**	**All respondents**	**Respondents reporting at least one violation**
Demographics		
Female	**58.1 (53.5–62.6)**	**66.6 (61.0–72.2)**
Male	**41.9 (37.4–46.5)**	**33.4 (27.8–39.0)**
Mean age in years	37.7 (36.3–39.1)	37.4 (35.4–39.3)
Mean household size	6.9 (6.3–7.5)	6.9 (6.1–7.7)
Marital status		
Married	70.8 (66.7–74.9)	73.1 (67.7–78.4)
Never married	18.8 (15.5–22.1)	16.8 (12.3–23.4)
Widowed	5.7 (3.9–7.4)	5.9 (3.5–8.3)
Divorced or separated	1.8 (0.9–2.7)	3.1 (1.3–4.9)
Other	2.8 (1.8–4.4)	1.4 (0.6–2.9)
Ethnic group (two-most frequently reported)		
Kikuyu	21.8 (16.5–27.0)	20.8 (13.3–28.3)
Luhya	**13.8 (11.1–16.5)**	**17.0 (12.6–21.3)**
Religion^b^		
Christian	87.6 (84.0–91.2)	92.6 (88.2–97.0)
Muslim	9.8 (6.9–12.6)	4.7 (0.5–8.9)
Other	3.4 (2.2–4.5)	3.6 (1.6–5.2)
Education/schooling		
Finished primary	20.9 (17.6–24.1)	21.0 (15.9–26.0)
Finished secondary	22.9 (18.9–26.9)	21.4 (15.6–27.2)
Finished tertiary	8.0 (5.2–10.8)	8.2 (4.8–11.6)
No education/schooling	11.8 (7.7–16.0)	11.0 (5.3–16.7)
Education/schooling interrupted by 2007 election	13.6 (10.0–17.2)	
Resumed after election	85.5 (75.3–95.8)	
Household owns land	56.7 (50.7–62.3)	53.8 (46.7–60.9)
Lost land as result of 2007 election	11.0 (8.2–13.8)	

Source: Study Database. Survey results are representative of the adult household-based population in Kenya in September 2011. ^a^All statistics are weighted percentages unless otherwise noted; the sum of column percentages for categorical variables (e.g., ethnic group) might exceed 100 due to rounding. ^b^Respondents were allowed to select more than one religion. Note: Column values in **bold** represent percentages for which there are statistically significant differences at or below the .05 level (adjusted Wald test of association used to test group differences) between “all respondents” and “respondents reporting at least one violation”. For a detailed version of the table, please see the Additional file 1.

### Sexual violence

Study results showed that 32.9% of women and 17.0% of men reported experiencing sexual violence in all time periods, and 26.3% of households reported at least one sexual violation. (Table 2) Reported incidents of sexual violence (per 1,000 persons/year) increased during the election violence period compared to pre-election for women (1671.8 vs. 33.3; p < .001) and men (695.9 vs. 5.7; p < .001). All forms of sexual violence (per 1,000 persons/year) increased during the election violence period compared to baseline, including sexual IPV (391.2 vs. 11.4; p < .001), politically-motivated sexual violence (1600.2 vs. 24.4; p < .001), and opportunistic sexual violence (183.3 vs. 5.2; p < .001), and were perpetrated by men and women. During the election violence period, the majority of male and female perpetrators of sexual violence were affiliated with government or political groups. The most commonly reported consequences of sexual violence included sexually transmitted infections, being bruised and beaten, anxiety and depression, bleeding, and stigmatization by family/community. Over half of the respondents believed that the election period sexual violence was politically-motivated (58.4%), and most respondents (67.4%) were unaware of the Kenya Sexual Offenses Act of 2006.

**Table 2:** Weighted means and rates of sexual violence: Kenyan adult household-based population (916 respondents)

**Characteristic**	**Weighted %**^**a **^**(95% CI)**		
Respondent households reporting sexual violence: all periods^b^	26.3 (21.6–30.9)		
*Female*	32.9 (26.9–38.9)		
*Male*	17.0 (12.0–22.0)		
	**Weighted mean occurrences per 1,000 persons/year**		
**Characteristic**	**Pre-2007 election**	**Election violence**^**c**^	**Since conclusion of election violence**^**d**^
**Reported sexual violence**	**39.1 (25.2–52.9)**	2370.1 (1528.5–3211.6)	**67.6 (42.4–92.8)**
*Female*	**33.3 (21.7–44.9)**	1671.8 (1082.0–2261.5)	**57.9 (34.4–81.5)**
*Male*	**5.7 (0.6–10.8)**	695.9 (295.1–1096.6)	**9.6 (3.6–15.5)**
Reported Sexual Intimate Partner Violence	**11.4 (7.8–15.0)**	391.2 (238.7–543.8)	**16.1 (10.9–21.3)**
*Female*	**10.6 (7.2–13.9)**	338.8 (213.2–464.4)	**14.8 (9.7–19.9)**
*Male*	**0.8 (0.0–1.7)**	52.4 (4.4–100.4)	**1.3 (0.0–2.6)**
Reported politically-motivated^e^ sexual violence	**24.4 (11.8–37.0)**	1600.2 (789.9–2410.5)	**38.9 (16.8–61.0)**
*Perpetrated by men only*	**17.9 (6.6–29.1)**	1267.2 (553.9–1980.4)	**30.9 (10.9–50.9)**
*Perpetrated by women only*	**5.7 (0.0–11.7)**	298.4 (0.0–600.0)	**5.3 (0.0–13.5)**
*Perpetrated by mixed-gender group*	3.5 (0.0–8.5)	215.2 (0.0–491.9)	4.4 (0.0–12.4)
Reported opportunistic sexual^f^ violence	**5.2 (2.3–8.1)**	183.3 (91.1–275.5)	**7.8 (4.4–11.3)**
*Perpetrated by men only*	**3.7 (1.3–6.2)**	109.8 (31.1–188.5)	**4.7 (1.9–7.6)**
*Perpetrated by women only*	**1.3 (0.0–2.6)**	36.0 (3.0–69.0)	**1.5 (0.0–3.2)**
*Perpetrated by mixed-gender group*	**0.9 (0.0–2.1)**	22.6 (0.0–47.6)	**1.4 (0.0–3.1)**
**Sexual violence by male perpetrators**	**38.7 (24.9–52.5)**	2140.9 (1310.7–2971.1)	**65.0 (39.8–90.1)**
*Affiliated with government or political group*	**18.2 (10.5–25.9)**	1506.2 (794.8–2217.7)	**27.3 (12.4–42.1)**
*Stranger unaffiliated with government or political group*	**2.2 (0.1–4.4)**	168.6 (57.3–279.9)	**4.2 (0.4–7.9)**
*No affiliation reported*	14.2 (4.0–24.4)	267.0 (0.0–854.5)	27.0 (8.1–45.9)
*Friend or known community member*	**1.0 (0.1–1.8)**	116.6 (21.3–211.8)	**1.9 (0.2–3.7)**
*Immediate family member*	**2.3 (0.2–4.5)**	60.5 (11.8–109.2)	**2.8 (0.1–5.6)**
*Intimate Partner*	**0.7 (0.0–1.4)**	21.0 (0.0–43.5)	**1.8 (0.3–3.3)**
**Sexual violence by female perpetrators**	**12.6 (4.2–20.9)**	584.8 (230.7–938.9)	**17.1 (5.7–28.6)**
*Affiliated with government or political group*	**6.9 (2.0–11.8)**	631.5 (86.9–1176.1)	**12.6 (1.6–23.5)**
*Stranger unaffiliated with government or political group*	**0.7 (0.0–1.7)**	70.2 (6.1–134.2)	**2.3 (0.0–4.6)**
*Immediate family member*	**2.2 (0.0–4.4)**	49.4 (3.1–95.7)	**2.2 (0.0–4.8)**
*Intimate Partner (spouse, boyfriend/girlfriend)*	**2.1 (0.7–3.5)**	47.9 (8.2–87.7)	**2.9 (0.6–5.2)**
*Friend or known community member*	0.6 (0.0–1.2)	23.1 (0.0–63.0)	1.1 (0.0–2.5)
*No affiliation reported*	0.6 (0.0–1.4)	13.9 (0.0–41.2)	1.2 (0.0–2.8)
**Self-reported consequences of sexual violence**	**Weighted %**^**a **^**(95% CI)**		
*Sexually Transmitted Infection*	**96.0 (91.8–100.0)**	98.5 (96.2–100.0)	**97.0 (90.9–100.0)**
*Bruised and beaten*	**79.2 (65.5–93.0)**	75.5 (57.6–93.5)	**70.2 (55.4–85.1)**
*Anxiety and depression*	**31.1 (15.1–47.4)**	45.1 (31.0–59.2)	**33.7 (16.3–51.1)**
*Bleeding*	**34.3 (21.1–47.5)**	38.2 (17.9–58.5)	**26.5 (17.4–35.6)**
*Stigmatized by family/community*	**15.0 (3.6–26.4)**	29.5 (11.0–48.1)	**11.5 (2.4–20.7)**
*Torn*	**14.7 (5.4–24.0)**	20.8 (5.7–35.9)	**16.8 (6.0–27.6)**
*Pregnant*	**10.3 (1.8–18.8)**	11.1 (0.7–21.6)	**9.7 (1.6–17.7)**
*Reproductive complications*	**11.9 (0.0–24.9)**	10.0 (0.0–21.9)	**5.3 (0.0–11.5)**
*Other*	**11.1 (1.0–21.1)**	19.1 (5.8–32.4)	**11.1 (4.3–17.9)**
**Characteristic**	**Weighted %**^**a **^**(95% CI)**		
Believes that sexual violence associated with 2007 election was politically-motivated	58.4 (54.1–62.6)		
Aware of Sexual Offenses Act of 2006	26.2 (21.9–31.2)		

Source: Study Database. Survey results are representative of the adult household-based population in Kenya in September 2011, as defined in Figure 1. ^a^All statistics are weighted percentages unless otherwise noted. ^b^Difference between “female” and “male” is statistically significant at the p < .001 level (adjusted Wald test of association used). ^c^Due to the 60-day period (January-March 1 2008) for which reported values were measured for election violence, it is possible for a mean during the election violence period to exceed 1,000; election violence includes “likely election violence”, which was determined if a respondent reported a sexual violation and did not report the period of occurrence, but reported the violation occurred in one of the following counties: Kiambu, Nairobi, Nakuru, Nandi, or Uashin Gishu. ^d^Defined as the period from March 2 2008 to administration of the survey in September 2011. ^e^Sexual violence occurring during a political circumstance, perpetrated by a political figure or state official, or in which political messages or ethnic slurs were uttered during an attack. ^f^Sexual violence that did not meet the criteria for politically-motivated violence. Note: Column values in **bold** represent mean occurrences for which there are statistically significant differences at or below the .05 level (Pairwise Chi-Square test used to test group differences) between “pre-2007 election” and “since conclusion of election violence”. For a detailed version of the table, please see the Additional file 1.

### Human rights violations and household morbidity

Fifty percent of households reported one or more human rights violations (including sexual violations) against family members, including physical, movement, and property violations as well as forced displacements during the election violence, with one-quarter of households reporting politically-motivated violence and a similar proportion of households also reporting opportunistic violence. Of households reporting violence, 10.9% reported the violations resulted in death. (Table 3)

**Table 3:** Weighted means and rates of human rights violations for household members of 916 adult Kenyan respondents

**Characteristic**	**Weighted %**^**a **^**(95% CI)**		
Respondent households reporting at least one violation^b^	50.0 (44.4–55.5)		
Respondent households reporting at least one physical violation	24.9 (20.0–29.7)		
Prevalence of households reporting violations^c^ that resulted in death	10.9 (7.9–14.0)		
	**Weighted mean occurrences per 1,000 persons/year**		
**Characteristic**	**Households experiencing violations prior to 2007 election**	**Households experiencing violations during election violence**^**d**^	**Households experiencing violations after election violence**^**e**^
Physical Violations	**25.0 (13.9–36.1)**	1987.1 (1269.2–2704.9)	**42.0 (23.5–60.5)**
Beating	**15.9 (9.9–21.9)**	1258.2 (789.7–1726.7)	**30.8 (16.0–45.5)**
Shot	**3.4 (0.0–7.4)**	172.5 (53.8–291.1)	**3.7 (0.0–9.1)**
Stabbed	**1.7 (0.3–3.0)**	126.9 (59.6–194.3)	**3.2 (0.4–6.0)**
Amputation	**1.1 (0.0–2.2)**	88.1 (17.1–159.0)	**1.0 (0.0–2.4)**
Other Unspecified Physical Assault	**3.0 (0.4–5.5)**	341.4 (170.8–512.1)	**3.3 (1.1–5.6)**
Prevalence of most commonly named perpetrators (by political or ethnic group affiliation) during election violence: Physical violations	**Weighted %**^**a **^**(95% CI)**		
Kalenjin	54.6 (38.9–70.3)		
Luo	19.5 (9.8–29.3)		
Orange Democratic Movement	15.2 (5.4–25.0)		
Kikuyu	11.2 (3.4–19.0)		
Party of National Unity	6.8 (1.0–12.7)		
**Characteristic**	**Weighted %**^**a **^**(95% CI)**		
How much human rights abuses by ethnic/political groups are something feared for self and family			
Extremely/quite a bit	64.1 (60.0–68.1)		
A little	13.9 (11.0–17.4)		
Not at all	21.9 (18.9–25.4)		
Felt coerced to vote in last (2007) election	4.9 (3.4–7.1)		
Feel safe to vote in future elections	74.8 (70.7–78.5)		

Source: Study Database. Survey results are representative of the adult household-based population in Kenya in September 2011, as defined in Figure 1. ^a^All statistics are weighted percentages unless otherwise noted. ^b^Includes physical and sexual violations. ^c^Includes sexual violations ending in death. ^d^Due to the 60-day period (January-March 1 2008) for which reported values were measured, it is possible for a mean during the election violence period to exceed 1,000; election violence includes “likely election violence”, which was determined if a respondent reported a violation and did not report the period of occurrence, but reported the violation occurred in one of the following counties: Kiambu, Nairobi, Nakuru, Nandi, or Uashin Gishu. ^e^Defined as the period from March 2 2008 to administration of the survey in September 2011. Note: Column values in **bold** represent mean occurrences for which there are statistically significant differences at or below the .05 level (Pairwise Chi-Square test used to test group differences) between “household experiencing violations prior to 2007 election” and “household experiencing violations after election violence”. For a detailed version of the table, please see the Additional file 1.

During the election violence period, overall physical violence incidents/1000 persons/year increased to 1987.1 compared to the pre-election time period (p < .001). Those occurrences decreased to 42.0 per 1,000 persons per day following the election violence period but did not reach pre-election levels.

The Kalenjin ethnic group was the most commonly reported perpetrator of physical violations (54.6%). Many respondents reported that they feared human rights abuses by ethnic/political groups for themselves or their family (64.1%); however, only 4.9% felt coerced to vote in the 2007 election and 74.8% felt safe to participate in future elections.

### Mental health outcomes

Among Kenyan adults, 36.5% met symptom criteria for MDD and 33.0% met symptom criteria for PTSD. (Table 4) Substance abuse was more common among male household members (males, 71.2%; females, 28.8%; p < .001). Symptoms of MDD and reported suicide attempts were higher among females (females, 63.3%; males, 36.7%; p = .01; and females, 68.5%; males, 31.5%; p = .04, respectively). Survivors of sexual violence were more likely to report suicidal ideation (sexual violence, 19.5%; no sexual violence, 8.2%; p = .01) and suicide attempt (sexual violence, 21.9%; no sexual violence, 8.4%; p=.002) than those not reporting sexual violence. Those who experienced forced displacement were more likely to report PTSD symptoms than those with no history of forced displacement (48.5% vs. 31.3%; p = .03).

**Table 4:** **Weighted prevalences of mental health outcomes: Kenyan adult household-based population, September 2011: 916 respondents**^**a**^

**Characteristic**	**Weighted % substance abuse (95% CI)**	**Weighted % MDD (95% CI)**	**Weighted % PTSD (95% CI)**	**Weighted % suicidal ideation (95% CI)**	**Weighted % suicide attempt (95% CI)**
Adults^b^	20.8 (16.6–25.0)	36.5 (31.2–41.8)	33.0 (27.8–38.3)	10.3 (7.7–13.0)	10.8 (8.3–13.3)
Female	**28.8 (21.6–36.0)**	**63.3 (54.4–72.3)**	60.7 (52.1–69.4)	66.1 (52.7–79.6)	**68.5 (58.7–78.3)**
Male	**71.2 (64.0–78.4)**	**36.7 (27.7–45.6)**	39.3 (30.6–47.9)	33.9 (20.4–47.3)	**31.5 (21.7–41.3)**
Sexual violence reported^c^	24.7 (15.7–33.5)	41.0 (27.0–55.0)	40.1 (28.6–51.6)	**19.5 (11.3–27.8)**	**21.9 (13.7–30.2)**
No sexual violence reported	19.8 (15.1–24.5)	35.0 (29.2–40.8)	30.9 (25.0–36.8)	**8.2 (5.4–11.0)**	**8.4 (5.9–10.9)**
Physical violence reported	26.6 (16.7–36.4)	38.8 (27.0–50.6)	40.4 (29.3–51.6)	13.5 (6.5–20.5)	13.1 (6.3–19.8)
No physical violence reported	19.6 (14.8–24.3)	35.9 (30.1–41.6)	31.0 (25.3–36.7)	9.7 (6.9–12.5)	10.3 (7.9–12.8)
Movement violations reported	27.1 (10.0–44.3)	43.0 (24.0–62.0)	**48.5 (34.3–62.7)**	14.9 (3.9–25.9)	16.0 (4.6–27.4)
No movement violations reported	20.2 (16.0–24.5)	35.7 (30.4–41.1)	**31.3 (25.8–36.9)**	10.0 (7.2–12.7)	10.4 (8.0–12.8)

Source: Study Database. Survey results are representative of the adult household-based population in Kenya in September 2011, as defined in Figure 1. ^a^All statistics are weighted percentages unless otherwise noted. ^b^**Bolded** values indicate statistically significant differences at or below the .05 level (adjusted Wald test of association used) between “female” and “male”. ^c^**Bolded** values from this point forward indicate statistically significant differences at or below the .05 level, calculated using a two-sample t-test for the difference between two proportions for each set of characteristics within each column. For a detailed version of the table, please see the Additional file 1.

Regarding mental health services, 36.8% of respondents reported inadequate access to mental healthcare (Table 5). Cost, fear of stigma, concerns about confidentiality, and access to a facility were the most commonly reported barriers. Over one-quarter of respondents identified mental health counseling as one of the most-needed services in addition to religious counseling/support groups. Males were more likely to state a need for income-generating projects (males; 42.2%, females; 30.5%; p = .04).

**Table 5:** Weighted health means and rates: Kenyan adult household-based population (916 respondents)

**Characteristic**	**Weighted %**^**a **^**(95% CI)**	
	**Female**	**Male**
Inadequate access to mental health care (self-reported)^b^	36.8 (30.8–42.8)	
Barriers to seeking mental health counseling (male and female)		
None	33.7 (28.9–38.8)	
Cost	30.4 (25.2–36.2)	
Fear of stigma	16.6 (13.3–20.6)	
Concerns about confidentiality	10.2 (8.2–12.8)	
Access to a program or facility	10.0 (7.3–13.5)	
Do not believe this would help	8.0 (5.9–10.8)	
Fear of community rejection or abandonment	3.3 (1.9–5.7)	
Fear of family rejection or abandonment	3.2 (2.0–5.1)	
Other	8.1 (4.2–11.7)	
Self-reported most needed services		
Religious counseling/support groups	74.2 (63.1–83.6)	65.3 (51.2–83.3)
Income-generating projects	**30.5 (25.1–35.9)**	**42.2 (32.7–51.8)**
Education	26.3 (20.1–32.5)	34.4 (26.3–42.5)
Mental health counseling	24.3 (18.7–30.0)	29.8 (21.8–37.7)
Medical assistance	20.5 (15.0–26.0)	17.4 (11.0–23.8)
Humanitarian assistance/food or shelter	11.4 (7.1–15.6)	18.0 (8.3–27.7)
Nothing	7.2 (4.5–9.9)	8.0 (3.8–12.2)
Other	15.7 (7.8–23.2)	22.8 (5.4–41.1)

Source: Study Database. Survey results are representative of the adult household-based population in Kenya in September 2011, as defined in Figure 1. ^a^All statistics are weighted percentages unless otherwise noted. ^b^If respondents reported no availability of counseling/support services in their area. Note: Column values in **bold** represent percentages for which there are statistically significant differences at or below the .05 level (adjusted Wald test of association used) for “female” and “male”. For a detailed version of the table, please see the Additional file 1.

## Discussion

Physical and sexual violence—both politically-motivated and opportunistic—increased during the 2007–2008 post-election period in Kenya. The post-election violence has been described as having both political and ethnic dimensions [2,3,17]. Our findings correlate to reports that indicate most of the attacks were carried out by Kalenjin pro-government PNU supporters and to a lesser degree by Kikuyu, Luhya and Luo [1-3]. With political parties aligned along ethnicity [1-3], the Luhya (ODM supporters)—commonly referred to as the *Luhya vote*—have historically been a deciding factor in the outcomes of elections since they tend to vote as a block and are a significant portion of the population [43-45]. In addition to retaliation for election violence against Kikuyu ethnicities [2], this might explain why more Luhya households were subjected to violence during the election violence of 2007–2008.

The post-election violence was reported to affect 136 constituencies in six of Kenya’s eight provinces [2,46]. Although our study did not assess the root causes of the post-election violence, the data suggest that land loss and the inability to resume schooling and/or vocational training, and unemployment—particularly among men—were associated with the violence. Some have suggested that unresolved deeply-rooted societal issues that have intensified since Kenya’s independence, including economic inequality, unemployment, structural imbalances, and land grievances, may have fueled the election-related violence [8,47].

The increase in sexual violence demonstrated in this study supports other findings [5,17,41] of widespread sexual violence in Kenya during the post-election period. Politically-motivated sexual violence was more commonly reported than opportunistic and might have been used to target specific groups due to political allegiance or ethnic background. However, our data also show that intimate partner and opportunistic violence increased ~35-fold during the election period. Unlike the sexual violence seen in conflicts [26,30], which is often characterized by gang rape, the sexual violence reported for Kenyan post-election sexual violence was more commonly single-person rape, molestation and genital mutilation overwhelmingly perpetrated by men and, to a lesser extent, women who were affiliated with a government or political group(s). The increase in genital mutilation during the election period supports media reports of ethnically motivated forced circumcision of males [48].

Despite the Sexual Offences Act of 2006 that provides law pertaining to victims and perpetrators of sexual violence in Kenya, there may be gaps in implementation. This study shows that the majority (68%) of Kenyans remain unaware of the Act. Survivors in many parts of Kenya lack access to formal justice and many areas use traditional court systems that do not recognize rape as a crime [49,50] and have police with limited training in the documentation of sexual violence [51,52]. Although a number of organizations in Kenya have provided essential services to survivors including healthcare, counseling, education and advocacy; care is centered primarily in Nairobi and lacking in mechanisms for data collection, analysis, forensic capacity, in addition to the application of results to programming and policy [18,53]. Furthermore, sexual violence is considered acceptable in some parts of the country, which fosters a culture of impunity and poses challenges for those seeking to report and prosecute cases of sexual violence [49,54,55].

Women who reported sexual violence were more likely to report substance abuse and suicide attempts than women not reporting sexual violence. Women were also more likely to report MDD symptoms compared to men. Men who reported sexual violence were more likely to report suicidal ideation and those who reported physical violence reported an increase in substance abuse (Additional file 1); a known risk for other mental health conditions [56,57]. Our study provides symptom prevalence data for MDD and PTSD. The findings are similar to other assessments that applied similar methodology and were conducted in communities affected by violent and protracted conflict [20-22,26]. However, there are few studies of MDD and PTSD prevalence in the adult Kenyan population for comparison. Our data highlight that mental health disorders, such as MDD, PTSD, suicidality, and substance use are important to address in Kenya. Mental health counseling was identified as the most-needed service by our respondents, and future mental health programming should address the reported obstacles to seeking mental health care such as cost, fear of stigma and confidentiality, and lack of access. Although there is evidence to support the integration mental health services in primary healthcare in Kenya, there is also a need for specialized and acute mental health services, especially following periods of violence [58].

### Limitations

Our findings represent the adult household-based population of Kenya and cannot be generalized to children under 18 years. The extent of differences between baseline and election-related violence might be affected by recall bias and methods used to assess timing of events. However, the 2007–2008 election period in Kenya is well recalled due to the severe violence. Literature on survey methods and cognitive psychology reports that events are recalled best if they are unique and/or traumatic, that traumatic and unique events are recalled over long periods of time, and that for these kinds of events a 10-year recall is considered reliable [29]. However, it is possible that events related to the election period may have been under- or over-estimated or misdated due to the reliance on respondent recall over a long period of time. Furthermore, given that a random household member was interviewed about violence, some respondents may have differing levels of knowledge of physical and/or sexual violence against other members of the household (based on position within the household, age differences, sex differences, etc.). This disclosure bias may affect the level of recall of violent events.

Although interviewers carefully explained that there would be no material or other gain by participation in the study, respondents might have exaggerated or underestimated responses if they believed doing so would be in their interest—especially given the highly political and ethnic nature of human rights reporting. Furthermore, although interviewers carefully explained confidentiality and maintained political neutrality in survey administration, questions about election-related violence—and in particular the reporting of perpetrators—might have been underreported if respondents were concerned that their responses might be shared with officials or affect their security in future elections, especially given the Kenyan Institutional Review Board requirement to have fingerprints or unidentifiable marks on survey consents. Ethnicity, sex, unfamiliarity with the interviewers, and other unidentified characteristics might have limited truthfulness of respondents to questions. Furthermore, although Kiswahili is the national language, survey administration may have been modified to meet the dialect in the surveyed region, which could have affected responses; however, our training process and supervision limited changes in the survey based on dialect.

Some extrapolated populations estimates are likely underestimates of the total number of people suffering violations (e.g., violations ending in death) since we could only interview surviving household members. Given displacement and movement of persons during the election violence, it is possible that at the time of the survey in a village there might have been double counting of episodes of violence if persons had moved in the interval period; however, our definition of household and training of data collectors to collect only household member information would have limited double counting and estimates of violence are more likely to be underreported. Given that the election-related violence anecdotally had regional patterns and was concentrated in certain areas of the country, cluster-sampling methodology might not have captured the extent of violence experienced in those areas; although population-based sampling revealed that the violence was not limited to certain regions and was more widespread than previously documented. Due to logistical restrictions in the field and sample size, the findings cannot be disaggregated by geographic regions or sub-grouped (i.e. by ethnicity) with confidence and are appropriate for estimating the prevalence at the national level only. The instruments used to measure symptoms of PTSD and MDD—although validated for use in studies—do not substitute for clinician-determined diagnoses, and thus, rates of PTSD and MDD symptoms should be interpreted with caution. Finally, the nature of this study (a multistage clustered random sample survey) allows for the determination of association of population characteristics, but not causality.

## Conclusion

Widespread violence during the 2007–2008 post-election period ensued in Kenya, including physical violence, sexual violence, and other human rights abuses perpetrated by both men and women. These findings emphasize the importance of assessing patterns of violence and perpetration among both men and women in studies of violence, and they reinforce that health systems and judicial and reparation frameworks should be comprehensive in addressing the needs of both men and women as survivors and perpetrators following periods of violence. A successful healthcare delivery strategy in Kenya will require specialized mental health programming and integration to meet the acute and long-term mental health needs of communities after the political violence. Finally, and despite efforts to investigate the post-election violence, there are concerns that justice has not been sufficiently pursued on a national level and that there has been minimal accountability for the post-election violence crimes [56,57]. These data can contribute to the evidence base informing ongoing efforts to secure justice for the victims of violence and restore peace among communities in Kenya.

## Competing interests

The funding organizations played no role in the design and conduct of the study, in the collection, management, analysis, and interpretation of the data, or in the presentation, review, or approval of the manuscript.

## Authors’ contributions

KJ designed the research study and survey instruments, oversaw the interviewer training, supervised data collection and data analysis, and wrote the initial draft of the manuscript. JS contributed to study design, survey development, interviewer training, data collection, data interpretation, and the initial draft of the manuscript. TS assisted in the initial grant proposal, supervised interviewers in the field, contributed to data interpretation, and drafting the initial manuscript. DN helped to inform the study design, contributed to interviewer training, and applied his expertise in the interpretation of data for the final manuscript. MK assisted with study design, conducted the data analysis, and was critical in the process of data interpretation. SR and SB supervised interviewers in the field and contributed to data interpretation and the initial manuscript. VM and AM assisted with interviewer training, pilot testing of the survey instrument, and data interpretation. DR assisted with data analysis and data interpretation. LL oversaw the study design and development of survey instruments, informed data collection, and supervised the data analysis and interpretation. All authors contributed to the writing of the manuscript. All authors read and approved the final manuscript.

## Supplementary Material

Additional file 1 **Detailed versions of Tables **1**, **2**, **3**, **4** and **5** are available as an additional file.** Click here for file
