# Supplementary material for: A national population-based assessment of 2007–2008 election-related violence in Kenya
Source: Confl Health. 2014 Jan 18;8:2. doi: 10.1186/1752-1505-8-2 (PMC3932993; doi:10.1186/1752-1505-8-2)
Supplement: Additional file 1 — Detailed versions of Tables 1, 2, 3, 4 and 5 are available as an additional file. [file 1752-1505-8-2-S1.doc]

**Additional file 1**

| **Table 1.** Weighted population characteristics: Kenyan adult household-based population, September 2011: 916 respondents. | | | |
| --- | --- | --- | --- |
|  | **Weighted %^a^ (95% CI)** | |  |
| **Characteristic** (respondents with characteristic/total respondents to question) | **All respondents** | **Respondents reporting at least one violation** | **p-value^b^** |
| Demographics |  |  |  |
| *Female (549/916; 318/462)* | 58.1 (53.5-62.6) | 66.6 (61.0-72.2) | **.04** |
| *Male (367/916; 144/462)* | 41.9 (37.4-46.5) | 33.4 (27.8-39.0) | **<.001** |
| *Mean age in years (916/916; 462/462)* | 37.7 (36.3-39.1) | 37.4 (35.4-39.3) | .39 |
| *Interquartile range for mean age in years* | 1^st^ quartile: 25.0  Median: 32.0  3^rd^ quartile: 43.0 | 1^st^ quartile: 26.0  Median: 32.0  3^rd^ quartile: 42.0 | -- |
| *Mean time residing in home village in years*  *(906/916; 453/462)* | 23.0 (21.2-24.8) | 22.2 (19.9-24.5) | .35 |
| *Mean household size (913/916; 460/462)* | 6.9 (6.3-7.5) | 6.9 (6.1-7.7) | .80 |
| *Mean no. of household members under 18*  *(902/916; 455/462)* | 2.6 (2.3-2.9) | 2.6 (2.3-3.0) | .78 |
| Marital status |  |  |  |
| *Married (649/916; 335/462)* | 70.8 (66.7-74.9) | 73.1 (67.7-78.4) | .21 |
| *Never married (167/916; 73/462)* | 18.8 (15.5-22.1) | 16.8 (12.3-23.4) | .21 |
| *Widowed (55/916; 32/462)* | 5.7 (3.9-7.4) | 5.9 (3.5-8.3) | .71 |
| *Divorced or separated (21/916; 16/462)* | 1.8 (0.9-2.7) | 3.1 (1.3-4.9) | .14 |
| *Other (25/916; 6/462)^c^* | 2.8 (1.8-4.4) | 1.4 (0.6-2.9) | .80 |
| Ethnic group |  |  |  |
| *Kikuyu (198/916; 92/462)* | 21.8 (16.5-27.0) | 20.8 (13.3-28.3) | .24 |
| *Luhya (118/916; 71/462)* | 13.8 (11.1-16.5) | 17.0 (12.6-21.3) | **.03** |
| *Kamba (86/916; 25/462)* | 10.4 (5.8-15.0) | 6.8 (2.8-10.8) | **.01** |
| *Luo (100/916; 54/462)* | 9.6 (5.9-13.2) | 10.9 (6.3-15.7) | .52 |
| *Kalenjin (87/916; 49/462)* | 9.1 (4.8-13.3) | 10.5 (5.2-15.9) | .30 |
| *Kisii (57/916; 40/462)* | 7.7 (3.1-12.2) | 9.6 (3.2-16.0) | .09 |
| *Other (268/916; 129/462)^d^* | 27.7 (22.1-33.2) | 24.4 (19.4-29.7) | .17 |
| Religion *^e^* |  |  |  |
| *Christian (816/911; 431/461)* | 87.6 (84.0-91.2) | 92.6 (88.2-97.0) | .14 |
| *Muslim (70/911; 15/461)* | 9.8 (6.9-12.6) | 4.7 (0.5-8.9) | .32 |
| *Other (31/911; 16/461)^f^* | 3.4 (2.2-4.5) | 3.6 (1.6-5.2) | .82 |
| Education/Schooling |  |  |  |
| *Finished primary (213/913; 105/460)* | 20.9 (17.6-24.1) | 21.0 (15.9-26.0) | .78 |
| *Finished secondary (205/913; 95/460)* | 22.9 (18.9-26.9) | 21.4 (15.6-27.2) | .22 |
| *Finished tertiary (62/913; 32/460)* | 8.0 (5.2-10.8) | 8.2 (4.8-11.6) | .95 |
| *No education/schooling (102/913; 54/460)* | 11.8 (7.7-16.0) | 11.0 (5.3-16.7) | .66 |
| Education/Schooling interrupted by 2007 election (70/566) | 13.6 (10.0-17.2) | |  |
| Resumed after election (56/70) | 85.5 (75.3-95.8) | |  |
| Household owns land (504/900; 236/454) | 56.7 (50.7-62.3) | 53.8 (46.7-60.9) | .17 |
| Lost land as result of 2007 election (95/910) | 11.0 (8.2-13.8) | |  |
| *Source: Study Database. Survey results are representative of the adult household-based population in Kenya in September 2011. ^a^All statistics are weighted percentages unless otherwise noted; the sum of column percentages for categorical variables (e.g., ethnic group) might exceed 100 due to rounding. Denominators are the sum of the survey weights for the respondents with the characteristic; number of respondents is given in the first column (as n=XX). ^b^Adjusted Wald test of association used; Pearson’s Chi-squared test used for continuous variables (e.g., mean household size). ^c^ Breakdown of “Other” marital status responses (all households and households reporting violence, respectively): Living with partner/unmarried (10), polygamous (8), husband missing (5), wife missing (2); living with partner/unmarried (2), polygamous (2), husband missing (1), wife missing (1). ^d^Breakdown of “Other” ethnicity response (all households) and households reporting violence, respectively: Ameru (39), Massai (29), Somali (27), Turkana (27), Giriama (21), Samburu (15), Taita (13), Tharaka (12), multiple ethnicities (8), Swahili (7), El-Molo (4), Sabaot (4), Mjibana (3), Embu (2), Ugandan (1), Sudanese (1), 55 respondents were of different or mixed ethnicities; Turkana (25), Ameru (22), Samburu (13), Massai (12), Tharaka (8), Giriama (6), Sabaot (4), Somali (4), El-Molo (3), Taita (3), Embu (2), Swahili (2); 25 respondents were of different or mixed ethnicities. ^e^Respondents were allowed to select more than one religion; column percentages therefore exceed 100. ^f^Breakdown of “Other” religion responses (all households and those reporting violence, respectively): Animist (21), Atheist (4), Jehovah’s Witness (1), Seventh-Day Adventist (2), not given (2), Hindu (1); Animist (11), Atheist (2), not given (2), Hindu (1).* | | | |

| **Table 2.** Weighted means and rates of sexual violence: Kenyan adult household-based population, September 2011: 916 respondents. | | | | | |
| --- | --- | --- | --- | --- | --- |
| **Characteristic** (respondents with characteristic/total respondents to question) | **Weighted %^a^ (95% CI)** | | | | |
| Respondent households reporting sexual violence: all periods (255/916) | 26.3 (21.6-30.9) | | | | |
| *Female (193/549)* | 32.9 (26.9-38.9) | | | | |
| *Male (62/367)* | 17.0 (12.0-22.0) | | | | |
| P-value (female and male) | **<.001** | | | | |
| *Mean no. sexual violations per household in survey area for households reporting sexual violence (694/253)* | 2.80 (2.49-3.11) | | | | |
|  | **Weighted mean occurrences per 1,000 persons/year** | | | |  |
| **Characteristic**  (n=number of occurrences of violation) | **Pre-2007 election** | **Election violence^b^** | | **Since conclusion of election violence^c^** | **p-value:**  **pre-2007 Election and Election Violence^d^** |
|  | (n=248) | (n=347) | | (n=276) |  |
| **Reported sexual violence (248, 347, 276)** | 39.1 (25.2-52.9) | 2370.1 (1528.5-3211.6) | | 67.6 (42.4-92.8) | **<.001** |
| *Female (209, 282, 222)* | 33.3 (21.7-44.9) | 1671.8 (1082.0-2261.5) | | 57.9 (34.4-81.5) | **<.001** |
| *Male (39, 105, 54)* | 5.7 (0.6-10.8) | 695.9 (295.1-1096.6) | | 9.6 (3.6-15.5) | **<.001** |
| Reported Sexual Intimate Partner Violence  (71, 68, 66) | 11.4 (7.8-15.0) | 391.2 (238.7-543.8) | | 16.1 (10.9-21.3) | **<.001** |
| *Female (66, 60, 61)* | 10.6 (7.2-13.9) | 338.8 (213.2-464.4) | | 14.8 (9.7-19.9) | **<.001** |
| *Male (5, 8, 5)* | 0.8 (0.0-1.7) | 52.4 (4.4-100.4) | | 1.3 (0.0-2.6) | **.002** |
| Reported politically-motivated^e^ sexual violence (138, 225, 137) | 24.4 (11.8-37.0) | 1600.2 (789.9-2410.5) | | 38.9 (16.8-61.0) | **<.001** |
| *Perpetrated by men only (105, 175, 104)* | 17.9 (6.6-29.1) | 1267.2 (553.9-1980.4) | | 30.9 (10.9-50.9) | **<.001** |
| *Perpetrated by women only (38, 37, 28)* | 5.7 (0.0-11.7) | 298.4 (0.0-600.0) | | 5.3 (0.0-13.5) | **.03** |
| *Perpetrated by mixed-gender group (25, 24, 20)* | 3.5 (0.0-8.5) | 215.2 (0.0-491.9) | | 4.4 (0.0-12.4) | .07 |
| Reported opportunistic sexual^f^ violence  (37, 36, 32) | 5.2 (2.3-8.1) | 183.3 (91.1-275.5) | | 7.8 (4.4-11.3) | **<.001** |
| *Perpetrated by men only (23, 23, 20)* | 3.7 (1.3-6.2) | 109.8 (31.1-188.5) | | 4.7 (1.9-7.6) | **.004** |
| *Perpetrated by women only (9, 7, 8)* | 1.3 (0.0-2.6) | 36.0 (3.0-69.0) | | 1.5 (0.0-3.2) | **.02** |
| *Perpetrated by mixed-gender group (7, 5, 7)* | 0.9 (0.0-2.1) | 22.6 (0.0-47.6) | | 1.4 (0.0-3.1) | **.04** |
| **Sexual violence by male perpetrators**  **(245, 343, 264)** | 38.7 (24.9-52.5) | 2140.9 (1310.7-2971.1) | | 65.0 (39.8-90.1) | **<.001** |
| *Affiliated with government or political group^g^ (108, 197, 92)* | 18.2 (10.5-25.9) | 1506.2 (794.8-2217.7) | | 27.3 (12.4-42.1) | **<.001** |
| *Stranger unaffiliated with government or political group (14, 25, 15)* | 2.2 (0.1-4.4) | 168.6 (57.3-279.9) | | 4.2 (0.4-7.9) | **.002** |
| *No affiliation reported (93, 92, 129)* | 14.2 (4.0-24.4) | 267.0 (0.0-854.5) | | 27.0 (8.1-45.9) | .20 |
| *Friend or known community member (8, 12, 7)* | 1.0 (0.1-1.8) | 116.6 (21.3-211.8) | | 1.9 (0.2-3.7) | **.01** |
| *Immediate family member (16, 12, 13)* | 2.3 (0.2-4.5) | 60.5 (11.8-109.2) | | 2.8 (0.1-5.6) | **.01** |
| *Intimate Partner (6, 5, 8)* | 0.7 (0.0-1.4) | 21.0 (0.0-43.5) | | 1.8 (0.3-3.3) | **.04** |
| **Sexual violence by female perpetrators**  **(84, 93, 80)** | 12.6 (4.2-20.9) | 584.8 (230.7-938.9) | | 17.1 (5.7-28.6) | **.001** |
| *Affiliated with government or political group^h^ (40, 63, 42)* | 6.9 (2.0-11.8) | 631.5 (86.9-1176.1) | | 12.6 (1.6-23.5) | **.01** |
| *Stranger unaffiliated with government or political group (4, 9, 7)* | 0.7 (0.0-1.7) | 70.2 (6.1-134.2) | | 2.3 (0.0-4.6) | **.02** |
| *Immediate family member (15, 10, 12)* | 2.2 (0.0-4.4) | 49.4 (3.1-95.7) | | 2.2 (0.0-4.8) | **.02** |
| *Intimate Partner (spouse, boyfriend/girlfriend; 15, 9, 13)* | 2.1 (0.7-3.5) | 47.9 (8.2-87.7) | | 2.9 (0.6-5.2) | **.01** |
| *Friend or known community member (5, 2, 3)* | 0.6 (0.0-1.2) | 23.1 (0.0-63.0) | | 1.1 (0.0-2.5) | .13 |
| *No affiliation reported (4, 3, 6)* | 0.6 (0.0-1.4) | 13.9 (0.0-41.2) | | 1.2 (0.0-2.8) | .16 |
| **Reported sexual violence types** |  |  | |  |  |
| *Rape (vaginal, oral, anal, object, unspecified; gang rape excluded; 30, 38, 30)* | 4.4 (1.9-6.8) | 244.2 (142.7-345.8) | | 9.4 (4.3-14.5) | **<.001** |
| *Molestation (26, 23, 29)* | 3.3 (1.5-5.2) | 130.3 (17.8-242.8) | | 5.3 (2.4-8.1) | **.01** |
| *Forced to perform act sexual assault against family member (8, 11, 9)* | 1.3 (0.3-2.4) | 74.4 (27.0-121.8) | | 2.3 (0.7-3.9) | **.001** |
| *Forced to undress (8, 11, 7)* | 1.1 (0.2-2.1) | 70.2 (0.0-144.0) | | 1.8 (0.3-3.3) | **.03** |
| *Genital mutilation (8, 6, 5)* | 1.0 (0.0-2.0) | 32.2 (0.0-68.5) | | 0.9 (0.0-2.0) | **.04** |
| *Rape in presence of others (1, 2, 1)* | 0.2 (0.0-0.6) | 12.9 (0.0-31.1) | | 0.4 (0.0-1.1) | **.08** |
| *Abduction (2, 2, 1)* | 0.2 (0.0-0.5) | 6.8 (0.0-19.0) | | 0.3 (0.0-0.8) | .14 |
| *Gang Rape (0, 1, 0)* | -- | 2.6 (0.0-7.8) | | -- | -- |
| *Forced to perform act sexual assault against non-family member (1, 1, 0)* | 0.02 (0.0-0.1) | 0.7 (0.0-2.0) | | -- | .16 |
| *Other^i^ acts (7, 13, 7)* | 1.1 (0.0-2.3) | 137.2 (0.0-337.3) | | 1.8 (0.2-3.3) | .09 |
| **Self-reported consequences of sexual violence** (n=number of respondents with characteristic/total respondents to question) | **Weighted %^a^ (95% CI)** | | | |  |
| *Sexually Transmitted Infection*  *(50/53, 50/52, 50/58)* | 96.0 (91.8-100.0) | | 98.5 (96.2-100.0) | 97.0 (90.9-100.0) | **<.001** |
| *Bruised and beaten (40/53, 42/52, 43/58)* | 79.2 (65.5-93.0) | | 75.5 (57.6-93.5) | 70.2 (55.4-85.1) | **.001** |
| *Anxiety and depression (18/53, 21/52, 22/58)* | 31.1 (15.1-47.4) | | 45.1 (31.0-59.2) | 33.7 (16.3-51.1) | **<.001** |
| *Bleeding (19/53, 21/52, 20/58)* | 34.3 (21.1-47.5) | | 38.2 (17.9-58.5) | 26.5 (17.4-35.6) | **.01** |
| *Stigmatized by family/community*  *(9/53, 12/52, 6/58)* | 15.0 (3.6-26.4) | | 29.5 (11.0-48.1) | 11.5 (2.4-20.7) | **<.001** |
| *Torn (8/53, 12/52, 9/58)* | 14.7 (5.4-24.0) | | 20.8 (5.7-35.9) | 16.8 (6.0-27.6) | **.01** |
| *Pregnant (6/53, 6/52, 7/58)* | 10.3 (1.8-18.8) | | 11.1 (0.7-21.6) | 9.7 (1.6-17.7) | **.01** |
| *Reproductive complications (5/53, 5/52, 4/58)* | 11.9 (0.0-24.9) | | 10.0 (0.0-21.9) | 5.3 (0.0-11.5) | **.01** |
| *Other^j^ (6/53, 7/52, 7/58)* | 11.1 (1.0-21.1) | | 19.1 (5.8-32.4) | 11.1 (4.3-17.9) | **.01** |
| **Characteristic** (n=number of respondents with characteristic/total respondents to question) | **Weighted %^a^**  **(95% CI)** | | | | |
| Believes that sexual violence associated with 2007 election was politically motivated |  | | | | |
| *Yes (507/892)* | 58.4 (54.1-62.6) | | | | |
| *No (280/892)* | 30.5 (27.0-34.1) | | | | |
| *Don’t know (105/892)* | 11.1 (9.3-13.3) | | | | |
| Aware of Sexual Offenses Act of 2006 |  | | | | |
| *Yes (214/887)* | 26.2 (21.9-31.2) | | | | |
| *No (621/887)* | 67.4 (63.1-71.5) | | | | |
| *Don’t know (52/887)* | 6.4 (4.6-8.8) | | | | |
| *Source: Study Database. Survey results are representative of the adult household-based population in Kenya in September 2011, as defined in Figure 1. ^a^All statistics are weighted percentages unless otherwise noted. Denominators are the sum of the survey weights for the respondents with the characteristic; number of respondents is given in the first column (as n=XX). ^b^Due to the 60-day period (January-March 1 2008) for which reported values were measured for election violence, it is possible for a mean during the election violence period to exceed 1,000; election violence includes “likely election violence,” which was determined if a respondent reported a sexual violation and did not report the period of occurrence, but reported the violation occurred in one of the following counties: Kiambu, Nairobi, Nakuru, Nandi, or Uashin Gishu. ^c^Defined as the period from March 2 2008 to administration of the survey in September 2011. ^d^Pairwise Chi-square test used. ^e^Sexual violence occurring during a political circumstance, perpetrated by a political figure or state official, or in which political messages or ethnic slurs were uttered during an attack. ^f^Sexual violence that did not meet the criteria for politically-motivated violence. ^g^Breakdown of groups for election period: unspecified state official or political leader (101), Kalenjin (15), Orange Democratic Movement (13), Kamba (6), Luo (4), Kisii (2), Massai (2), Sabaot Land Defence Force (2), Kikuyu (1), Orange Democratic Movement—Kenya (1). ^h^Breakdown of groups for election period: unspecified state official or political leader (42), Kalenjin (12), Orange Democratic Movement (7), Kisii (2), Samburu (2). ^i^Breakdown of “Other” for election violence period: forced marriage (4); remaining cases (for all periods) were unspecified. ^j^Breakdown of “Other” for election violence period: “anguish” (1); “pain” (2), “economic” consequences (1); remaining cases (for all periods) were unspecified.* | | | | | |

| **Table 3.** Weighted means and rates of human rights violations for household members of 916 adult Kenyan respondents, September 2011. | | | | | |
| --- | --- | --- | --- | --- | --- |
| **Characteristic** (households with characteristic/total respondents to question) | | | **Weighted %^a^ (95% CI)** | | |
| *Respondent households reporting at least one violation^b^ (462/916)* | | | 50.0 (44.4-55.5) | | |
| *Mean no. of household member deaths due to 2007 election violence (184/864)* | | | 0.21 (0.13-0.29) | | |
| *Mean no. of household members injured due to 2007 election violence (266/832)* | | | 0.30 (0.20-0.39) | | |
| *Respondent households reporting at least one physical violation (222/916)* | | | 24.9 (20.0-29.7) | | |
| *Prevalence of households reporting violations among those under 18 years (78/916)* | | | 8.3 (5.8-10.8) | | |
| *Prevalence of households reporting violations^c^ that resulted in death (102/916)* | | | 10.9 (7.9-14.0) | | |
| *Mean violations per household in survey area for households that have experienced a violation (814/398)* | | | 2.03 (1.86-2.21) | | |
|  | **Weighted mean occurrences per 1,000 persons/year** | | | |  |
| **Characteristic**  (n=number of occurrences of violation) | **Households experiencing violations prior to 2007 election** | **Households experiencing violations during election violence^d^** | | **Households experiencing violations after election violence^e^** | **p-value:**  **pre-2007 Election and Election Violence^f^** |
|  | (n=343) | (n=627) | | (n=211) |  |
| Physical Violations (154, 280, 160) | 25.0 (13.9-36.1) | 1987.1 (1269.2-2704.9) | | 42.0 (23.5-60.5) | **<.001** |
| *Beating (102, 171, 116)* | 15.9 (9.9-21.9) | 1258.2 (789.7-1726.7) | | 30.8 (16.0-45.5) | **<.001** |
| *Shot (17, 30, 14)* | 3.4 (0.0-7.4) | 172.5 (53.8-291.1) | | 3.7 (0.0-9.1) | **.002** |
| *Stabbed (10, 23, 10)* | 1.7 (0.3-3.0) | 126.9 (59.6-194.3) | | 3.2 (0.4-6.0) | **<.001** |
| *Amputation (6, 12, 4)* | 1.1 (0.0-2.2) | 88.1 (17.1-159.0) | | 1.0 (0.0-2.4) | **.01** |
| *Other Unspecified Physical Assault (19, 44, 16)* | 3.0 (0.4-5.5) | 341.4 (170.8-512.1) | | 3.3 (1.1-5.6) | **<.001** |
| Movement Violations (64, 132, 30) | 9.5 (4.6-14.5) | 1020.7 (541.0-1500.4) | | 11.5 (3.2-19.7) | **<.001** |
| *Forced Displacement (54, 119, 28)* | 7.5 (3.7-11.3) | 916.4 (457.9-1374.8) | | 11.0 (2.7-19.2) | **<.001** |
| *Capture/abduction (10, 13, 2)* | 2.0 (0.1-4.0) | 104.3 (23.8-184.9) | | 0.5 (0.0-1.2) | **.01** |
| Property theft/destruction (48, 111, 51) | 6.9 (3.4-10.5) | 763.3 (459.8-1066.8) | | 15.1 (7.1-23.1) | **<.001** |
| Threatened (6, 10, 2) | 0.7 (0.0-1.7) | 53.6 (0.0-114.1) | | 0.7 (0.0-1.4) | **.04** |
| Other^g^ Violations (10, 24, 7) | 0.9 (0.1-1.7) | 192.7 (0.0-399.7) | | 1.7 (0.2-3.3) | **.03** |
| Prevalence of most commonly named perpetrators (by political or ethnic group affiliation) during election violence: Physical violations (n=number of respondents with characteristic/total respondents to question) | **Weighted %^a^**  **(95% CI)** | | | | |
| *Kalenjin (45/88)* | 54.6 (38.9-70.3) | | | | |
| *Luo (15/88)* | 19.5 (9.8-29.3) | | | | |
| *Orange Democratic Movement (11/88)* | 15.2 (5.4-25.0) | | | | |
| *Kikuyu (9/88)* | 11.2 (3.4-19.0) | | | | |
| *Party of National Unity (7/88)* | 6.8 (1.0-12.7) | | | | |
| **Characteristic** (n=number of respondents with characteristic/total respondents to question) | **Weighted %^a^**  **(95% CI)** | | | | |
| How much human rights abuses by ethnic/political groups are something feared for self and family |  | | | | |
| *Extremely/quite a bit (554/878)* | 64.1 (60.0-68.1) | | | | |
| *A little (128/878)* | 13.9 (11.0-17.4) | | | | |
| *Not at all (196/878)* | 21.9 (18.9-25.4) | | | | |
| Felt coerced to vote in last (2007) election |  | | | | |
| *Yes (47/863)* | 4.9 (3.4-7.1) | | | | |
| *No (804/863)* | 93.5 (90.7-95.4) | | | | |
| *Don’t know (12/863)* | 1.6 (0.9-3.0) | | | | |
| Feel safe to vote in future elections |  | | | | |
| *Yes (676/894)* | 74.8 (70.7-78.5) | | | | |
| *No (192/894)* | 21.7 (18.3-25.4) | | | | |
| *Don’t know (26/894)* | 3.5 (2.3-5.3) | | | | |
| What would help respondent to feel safe in the future |  | | | | |
| *Increased police presence (195/423)* | 47.1 (41.8-52.4) | | | | |
| *Justice against attacker(s) (119/423)* | 30.2 (22.8-38.5) | | | | |
| *Change in attitude/education of men (91/423)* | 19.6 (15.1-25.1) | | | | |
| *Change in attitude/education of women (68/423)* | 15.6 (12.2-19.8) | | | | |
| *Enforcement of laws against sexual violence (53/423)* | 13.4 (9.7-18.1) | | | | |
| *Nothing (29/423)* | 8.2 (5.4-12.1) | | | | |
| *Don’t know (29/423)* | 5.5 (3.4-8.6) | | | | |
| *Other^h^ (88/423)* | 21.1 (16.3-26.9) | | | | |
| What has helped respondent through experiences |  | | | | |
| *Religion (166/358)* | 43.9 (38.4-49.6) | | | | |
| *Discussions with family members/friends (240/358)* | 69.8 (54.2-87.1) | | | | |
| *Taking care of household (19/358)* | 5.8 (4.1-8.1) | | | | |
| *Discussions with other survivors of sexual violence (16/358)* | 5.5 (3.0-10.1) | | | | |
| *Assistance from NGO workers (23/358)* | 5.3 (3.6-7.7) | | | | |
| *Trying to forget about it (17/358)* | 5.3 (3.1-9.0) | | | | |
| *Not telling about the incident (16/358)* | 5. 0 (2.2-10.8) | | | | |
| *Other^i^ (59/358)* | 17.5 (4.3-30.8) | | | | |
| *Source: Study Database. Survey results are representative of the adult household-based population in Kenya in September 2011, as defined in Figure 1. ^a^All statistics are weighted percentages unless otherwise noted. Denominators are the sum of the survey weights for the respondents with the characteristic; number of respondents is given in the first column (as XX). ^b^Includes physical and sexual violations. ^c^Includes sexual violations ending in death. ^d^Due to the 60-day period (January-March 1 2008) for which reported values were measured, it is possible for a mean during the election violence period to exceed 1,000; election violence includes “likely election violence,” which was determined if a respondent reported a violation and did not report the period of occurrence, but reported the violation occurred in one of the following counties: Kiambu, Nairobi, Nakuru, Nandi, or Uashin Gishu. ^e^Defined as the period from March 2 2008 to administration of the survey in September 2011. ^f^Pairwise Chi-square test used. ^g^Breakdown for “Other” during election violence: verbal abuse (3), detention (1), left unconscious (1), burned (2), unspecified torture (1); remainder of other violations is unspecified for all periods. ^h^Breakdown of “Other:” employment (7), peace (11), ending tribalism (12), prayer (7), ending corruption (8), increased in government stability (5), unspecified (38). ^i^Breakdown of “Other:” work/job/employment (9), medical care provider (7), reporting to police/community policing (3), seeking or being able to forgive (2), village elders/teachers (2), personal interests (2), healing ceremonies (1), unspecified (11), 22 respondents answered this question but reported that they did not experience sexual violence.* | | | | | |

| **Table 4.** Weighted prevalences of mental health outcomes: Kenyan adult household-based population, September 2011: 916 respondents.^a^ | | | | | |
| --- | --- | --- | --- | --- | --- |
| **Characteristic**  (respondents with characteristic/total respondents to question) | **Weighted % substance abuse**  (95% CI) | **Weighted % MDD**  (95% CI) | **Weighted %**  **PTSD**  (95% CI) | **Weighted % suicide ideation**  (95% CI) | **Weighted % suicide attempt**  (95% CI) |
| Adults  (183/916, 212/597, 186/577, 94/889, 93/906) | 20.8 (16.6-25.0) | 36.5 (31.2-41.8) | 33.0 (27.8-38.3) | 10.3 (7.7-13.0) | 10.8 (8.3-13.3) |
| *Female*  *(52/183, 139/212, 119/186, 65/94, 62/93)* | **28.8 (21.6-36.0)** | **63.3 (54.4-72.3)** | 60.7 (52.1-69.4) | 66.1  (52.7-79.6) | **68.5**  **(58.7-78.3)** |
| *Male*  *(131/183, 73/212, 67/186, 29/94, 31/93)* | **71.2 (64.0-78.4)** | **36.7 (27.7-45.6)** | 39.3 (30.6-47.9) | 33.9  (20.4-47.3) | **31.5**  **(21.7-41.3)** |
| ***p-value^b^*** | **<.001** | **.01** | .20 | .17 | **.04** |
| *Sexual violence reported*  *(36/162, 49/162, 53/162, 30/162, 29/162)* | 24.7 (15.7-33.5) | 41.0 (27.0-55.0) | 40.1 (28.6-51.6) | **19.5**  **(11.3-27.8)** | **21.9**  **(13.7-30.2)** |
| *No sexual violence reported*  *(141/730, 157/730, 127/730, 60/730, 59/730)* | 19.8 (15.1-24.5) | 35.0 (29.2-40.8) | 30.9 (25.0-36.8) | **8.2 (5.4-11.0)** | **8.4 (5.9-10.9)** |
| ***p-value^c^*** | .34 | .44 | .52 | **.01** | **.002** |
| *Physical violence reported*  *(40/160, 50/160, 53/160, 22/160, 17/160)* | 26.6 (16.7-36.4) | 38.8 (27.0-50.6) | 40.4 (29.3-51.6) | 13.5 (6.5-20.5) | 13.1 (6.3-19.8) |
| *No physical violence reported*  *(143/756, 162/756, 133/756, 72/756, 76/756)* | 19.6 (14.8-24.3) | 35.9 (30.1-41.6) | 31.0 (25.3-36.7) | 9.7 (6.9-12.5) | 10.3 (7.9-12.8) |
| ***p-value*** | .21 | .66 | .14 | .32 | .46 |
| *Movement violations reported*  *(17/65, 25/65, 28/65, 10/65, 10, 65)* | 27.1 (10.0-44.3) | 43.0 (24.0-62.0) | **48.5 (34.3-62.7)** | 14.9 (3.9-25.9) | 16.0 (4.6-27.4) |
| *No movement violations reported*  *(166/851, 187/851, 158/851, 84/851, 83/851)* | 20.2 (16.0-24.5) | 35.7 (30.4-41.1) | **31.3 (25.8-36.9)** | 10.0 (7.2-12.7) | 10.4 (8.0-12.8) |
| ***p-value*** | .44 | .47 | **.03** | .40 | .34 |
| Female |  |  |  |  |  |
| *Sexual Violence Reported*  *(19/119, 41/119, 42/119, 22/119, 23/119)* | **17.8 (10.5-25.0)** | 45.2 (29.5-60.9) | 41.2 (27.4-54.9) | 17.7 (8.7-26.7) | **23.0**  **(13.8-32.2)** |
| *No Sexual Violence Reported*  *(31/416, 95/416, 74/416, 41/416, 36/316)* | **8.3 (4.6-11.9)** | 37.5 (29.9-45.0) | 33.0 (25.4-40.5) | 10.1 (6.2-14.0) | **9.8 (6.3-13.3)** |
| ***p-value*** | **.02** | .39 | .30 | .13 | **.01** |
| *Physical violence reported*  *(14/108, 37/108, 38/108, 15/108, 12/108)* | 12.8 (5.9-19.7) | 43.0 (29.5-56.5) | 44.4 (32.3-56.4) | 13.2 (4.1-22.4) | 15.1 (7.2-23.1) |
| *No physical violence reported*  *(38/441, 102/441, 81/441, 50/441, 50/443)* | 9.7 (5.6-13.8) | 30.1 (31.9-46.3) | 32.7 (25.0-40.3) | 11.6 (8.0-15.2) | 12.2 (9.1-15.4) |
| ***p-value*** | .45 | .62 | .11 | .75 | .50 |
| *All forms of violence reported*  *(22/162, 53/162, 52/162, 94/162, 26/162)* | 14.1 (8.2-20.1) | 44.3 (32.0-56.5) | 39.9 (29.1-50.8) | **18.1**  **(10.4-25.8)** | **19.4**  **(12.4-26.5)** |
| *No violence reported*  *(30/187, 86/387, 67/387, 38/387, 36/387)* | 8.7 (4.8-12.6) | 37.8 (30.0-45.6) | 14.1 (8.2-20.1) | **9.4 (5.8-13.0)** | **10.1 (6.5-13.7)** |
| ***p-value*** | .14 | .38 | .34 | **.05** | **.02** |
| Male |  |  |  |  |  |
| *Sexual Violence Reported*  *(17/131, 8/43, 11/43, 8/43, 7/43)* | 44.3 (22.1-66.5) | 26.4 (5.1-47.6) | 37.3 (16.7-58.0) | **25.1 (7.0-43.2)** | 18.8 (6.6-31.0) |
| *No Sexual Violence Reported*  *(110/314, 62/314, 53/314, 19/314, 23/314)* | 34.0 (25.9-42.1) | 32.1 (23.5-40.7) | 28.6 (21.1-36.1) | **5.9 (2.2-9.7)** | 6.8 (3.7-9.9) |
| ***p-value*** | .39 | .63 | .44 | **.04** | .06 |
| *Physical violence reported*  *(26/52, 13/52, 15/52, 7/52, 5/52)* | **55.0 (35.3-74.7)** | 31.1 (13.0-49.3) | 33.0 (16.2-49.8) | 14.0 (1.7-26.2) | 9.0 (0.0-19.1) |
| *No physical violence reported*  *(105/315, 50/315, 52/315, 22/315, 26/315)* | **32.3 (24.0-40.5)** | 31.8 (23.1-40.4) | 29.1 (21.5-36.7) | 7.3 (3.2-11.4) | 7.9 (4.6-11.2) |
| ***p-value*** | **.04** | .95 | .68 | .31 | .84 |
| *All forms of violence reported*  *(29/71, 15/71, 17/71, 8/71, 8/71)* | 44.5 (26.7-62.2) | 28.8 (12.7-44.8) | 31.2 (16.6-45.7) | 13.9 (2.9-24.9) | 10.3 (1.6-18.9) |
| *No violence reported (102/296, 58/296, 21/296, 19/29, 23/296)* | 33.3 (24.7-42.0) | 32.4 (23.6-41.2) | 29.4 (21.7-37.4) | 7.0 (2.9-11.1) | 7.6 (4.2-11.0) |
| ***p-value*** | .23 | .70 | .83 | .25 | .57 |
| *Source: Study Database. Survey results are representative of the adult household-based population in Kenya in September 2011, as defined in Figure 1. ^a^All statistics are weighted percentages unless otherwise noted. Denominators are the sum of the survey weights for the respondents with the characteristic. ^b^Adjusted Wald test of association used. ^c^P-values from this point forward calculated using a two-sample t-test for the difference between two proportions.* | | | | | |

| **Table 5.** Weighted health means and rates: Kenyan adult household-based population, September 2011: 916 respondents. | | | |
| --- | --- | --- | --- |
|  | **Weighted %^a^ (95% CI)** | |  |
| **Characteristic** (f=female respondents with characteristic/total female respondents to question, m=male respondents with characteristic/total male respondents to question) | **Female** | **Male** | **p-value^b^** |
| **Respondent Health** |  |  |  |
| Substance use |  |  |  |
| *Never used drugs or alcohol (f=433/517, m=185/353)* | 81.8 (76.8-86.9) | 52.8 (45.2-60.5) | **<.001** |
| *Current substance abuser (f=52/543, m=133/366)* | 10.4 (6.9-13.8) | 35.3 (27.7-42.9) | **.05** |
| *Intake increased since 2007 election violence (f=9/517, m=14/353)* | 1.6 (0.5-2.7) | 4.5 (1.7-7.3) | .41 |
| Mental Health (self-reported) |  | | |
| *Inadequate access to mental health care ^c^ (346/893)* | 36.8 (30.8-42.8) | | |
| *Mental health counseling received since 2007 election violence (f=40/169, m=27/107)* | 27.6 (19.1-36.1) | 27.3 (16.6-38.1) | .96 |
| Barriers to seeking mental health counseling (male and female) |  | | |
| *None (294/847)* | 33.7 (28.9-38.8) | | |
| *Cost (269/847)* | 30.4 (25.2-36.2) | | |
| *Fear of stigma (133/847)* | 16.6 (13.3-20.6) | | |
| *Concerns about confidentiality (81/847)* | 10.2 (8.2-12.8) | | |
| *Access to a program or facility (84/847)* | 10.0 (7.3-13.5) | | |
| *Do not believe this would help (58/847)* | 8.0 (5.9-10.8) | | |
| *Fear of community rejection or abandonment (22/847)* | 3.3 (1.9-5.7) | | |
| *Fear of family rejection or abandonment (26/847)* | 3.2 (2.0-5.1) | | |
| *Other^d^ (64/847)* | 8.1 (4.2-11.7) | | |
| Self-reported most needed services |  | | |
| *Religious counseling/support groups (f=375/509, m=219/340)* | 74.2 (63.1-83.6) | 65.3 (51.2-83.3) | .35 |
| *Income-generating projects (f=169/509, m=136/340)* | 30.5 (25.1-35.9) | 42.2 (32.7-51.8) | **.03** |
| *Education (f=129/509, m=110/340)* | 26.3 (20.1-32.5) | 34.4 (26.3-42.5) | .10 |
| *Mental health counseling (f=121/509, m=94/340)* | 24.3 (18.7-30.0) | 29.8 (21.8-37.7) | .21 |
| *Medical assistance (f=98/509, m=55/340)* | 20.5 (15.0-26.0) | 17.4 (11.0-23.8) | .38 |
| *Humanitarian assistance/food or shelter (f=64/509, m=59/340)* | 11.4 (7.1-15.6) | 18.0 (8.3-27.7) | .21 |
| *Nothing (f=36/509, m=25/340)* | 7.2 (4.5-9.9) | 8.0 (3.8-12.2) | .73 |
| *Other^e^ (f=81/509, m=75/340)* | 15.7 (7.8-23.2) | 22.8 (5.4-41.1) | .47 |
| *Source: Study Database. Survey results are representative of the adult household-based population in Kenya in September 2011, as defined in Figure 1. ^a^All statistics are weighted percentages unless otherwise noted. Denominators are the sum of the survey weights for the respondents with the characteristic; number of respondents is given in the first column (as n=XX). ^b^Adjusted Wald test of association used. ^c^If respondents reported no availability of counseling/support services in their area. ^d^Breakdown of “Other:” interference with responsibilities at home or work (26), distance (4), fear for physical security (4), lack of time (3), not knowing where to obtain counseling (1), lack of services or infrastructure (2), unspecified (24). ^e^Breakdown of “Other:” Skills training (57), traditional ceremonies (36), traditional healer (23), government intervention (3), not given (30); 7 respondents answered this question and reported that they were not affected by violence.* | | | |
